# The Management of a Dental Practice*Note—We reprint this article from The Journal, which is the organ of “true professionalism,” and it may seem to our readers to be a close copying of the ideas which underlie the much-criticised trade combines, or department store methods. In this case a good professional man heads a successful practice.

**Published:** 1907-07-15

**Authors:** Edward C. Briggs


### THE MANAGEMENT OF A DENTAL PRACTICE.*

BY EDWARD C. BRIGGS, M.D., D.M.D.

The writer disclaims all responsibility in presenting this paper to the American Academy of Dental Science. Your Executive Committee chose the title and one of its members, Dr. Cutter, kindly suggested the subjects to be treated. If, therefore, in stating what I do in the conduct of my office the personal pronoun seems prominent, please blame the committee for the egotism, and not me. It does not appear that I am expected to discuss the management of patients themselves, and as a matter of fact, it seems to me that that subject, complicated as it is, can be dismissed in a few words. If you will "put yourself in his place," sincerely and truthfully, all things will be made plain before you. *You* would not want instruments of questionable cleanliness put into *your* mouth. You would not want to put your head on a napkin where another's head had been. You would not want to be in doubt whether the hands going to your mouth had been carefully washed after the previous patient. Nor would you like to see the operator stroke his moustache and then put his fingers unwashed into your mouth. If a patient seems unreasonable and is fretting over some condition which has arisen, don't stand on what you are pleased to call your dignity, and end by losing your temper and your patient. Stop and think how it would seem to you if you had the patient's point of view. Admit all the patient's contentions that you can, and honestly do all that is possible to bring about a happy result. The end is harmony—the patient has renewed loyalty, and you get the credit of being a tactful man.

---

*NOTE—We reprint this article from *The Journal*, which is the organ of "true professionalism," and it may seem to our readers to be a close copying of the ideas which underlie the much-criticised trade combines, or department store methods. In this case a good professional man heads a successful practice.

And now to the business arrangements: I will first speak of appointments.

Appointments are made for myself and six associates by one secretary. If a patient calls for an appointment, he is asked if he is suffering; if so, he is attended to by the one who is at leisure or who has the first free time. Of course, every one is anxious to find time, because that first visit may lead to the patient becoming permanently attached to the dentist who sees him.

If there is no special hurry, the patient is given a half hour for examination. This half hour may be with the one he asks to have, or, as is often suggested, the appointment may be with some one who has the first free time, who will examine and report to the doctor who was first inquired for.

If I were the one receiving the report, and found it impossible to attend to the patient in a reasonably short time, it then becomes much easier to persuade the patient to go to the one who has just examined him than to go to an absolute stranger.

The appointments are made in a large diary, one page for each day of the year, and the patient is given a card—sample shown—not unlike a visiting card, with the day, date and hour of appointment written on it.

If wedging is necessary, such notice is written on the card with a date, four days previous to the appointment, on which the patient is to come for the wedge.

Regular patients, that is, those who have been seen before and are to be looked after at regular intervals, are given one, two, three or six months ahead, as their cases demand, two or three (the most) appointments; the first one of a half hour and the other two one hour or possibly one and one- half hours. In the first—of half an hour—the teeth are examined and any scaling and polishing done that the time admits of Patients requiring a set piece of work like crown or bridge work, are given what is known as a "group," "simple group" for a crown, "double group" for a bridge, and the appointment secretary, on being told, arranges the appointments close together, but with due allowance between for the laboratory work and the length of the appointments according to a prearranged system.

Patients having made appointments three, six or more months ahead are, at their request, notified one week before their sittings.

Unless a special group of appointments is evidently necessary, no patient is allowed to engage more than three sittings in advance.

The engagement of time ahead by regular patients often fills up the time, even whole months, completely, thus not leaving space for the new or unexpected cases. These are provided for by keeping what is called the "emergency list," and when, as is sure to happen, engagements are cancelled, the time is filled from the emergency list.

Patients suffering from unexpected pain or disaster, are attended by some associate who has free time, or, if absolutely demanding his own special dentist, the time is made for him by putting off some one who will will not suffer by delay. It is the absolute rule of the office that any one in suffering takes precedence over all others, and any one whose time is taken away from him for such an emergency, who rebels, is politely told that we consider the relief of suffering humanity our first duty, and if he, the rebellious one, does not appreciate the justice and equity of that position, he can find some other dentist.

### ASSOCIATES.

In receiving an associate into my office, the aim has been to so arrange that he shall feel perfectly independent and realize that aside from the fact that I am the elder member of the association, and the medium through which patients come, he is in all full respects as free as I. As a matter of fact, he is perhaps more independent than I, since I am his agent in the matter of running the office, collecting the bills, hiring and discharging the employees and being the target for such patients as are in any way dissatisfied. He receives the labor of all the employees in common with his other associates. He equips his own operating room and purchases his own materials. He pays for all his privileges by paying to me as the owner of the office and the creator of the practice a per cent. of the cash he receives for his work. If no cash, then no payment, and if absent on acccount of illness or in the pursuit of pleasure there is no account for rent piling up against him. He is under no contract to remain, and only by mutual agreement can I close my dealings with him.

The percentage paid me by him is adjusted on a sliding scale, so much for a certain length of time, then a lesser amount for another period, then another reduction, and so on until at length he reaches a per cent, which in my judgment and that of business men whom I have consulted, is the proper amount to be set aside from one's gross income to run one's business. As the exact percentages is amatter which concerns my associates, I am not at liberty to state them. There is no doubt that associates who have been with me long enough to possess a full practice could take that practice and go to less expensive quarters, but I have no doubt they recognize, and recognize truly, that they could not accomplish as much work in a given time, or retain so good a clientele when not providing such excellent service and equipment.

My associates can call on me at all times for consultation.,

I make no pretense to being a superior operator to any of my associates. In fact, in referring patients to them, I am often able to say in reference to some particular piece of work, that it will be done better than I can do it. I do maintain, however, and all of my confreres of the same number of years of practice will, I know, agree, that experience does give something that can be obtained in no otherway, and a man of twenty to thirty years of experience who has not something to give to the man of five, ten or fifteen years has lived in vain and to no purpose.

### EMPLOYEES.

As there is a goodly number of us associates—"we are seven"—there is, of course, a number of employees, but in my description of their work, bear in mind that the system has always been the same, although when there were fewer associates, there were fewer employees.

At present the corps consists of three secretaries, four chair assistants, two laboratory assistants, a housekeeper, a janitor, a laundress and a door-boy.

One secretary has charge of all the appointments, and there is no idle moment for her. There were at the time of writing this paper———definite engagements on the seven books, to say nothing of the emergency lists. The arrangement of these, the notifications to patients, the constant changes, the finding of time for sudden calls, et cetera, keep one woman very busy.

One secretary keeps the books and sends out the bills. The accounts are kept by the card system and in double entry. The billheads bear the names of the seven associates, but the cheques are made payable to the writer.

A statement of all unpaid accounts on the books is rendered the first of every month. To this system which the writer has pursued since the beginning of his practice is credited a somewhat remarkable immunity from so-called "bad accounts." This rule is so absolute that a patient who contracts a debt on the 30th of a month and has an appointment for the 1st of the following month finds in his morning mail of that first day a statement of his debt of the 30th ultimo.

This arrangement keeps a patient in touch with his account. He does not easily forget how often he has been treated or how much has been done. If he is a good business man he is glad to pay as he goes. If one of limited means, he finds it easier to pay a little at a time, and not have at the end of three or six months a bill so large as to be difficult of attention. If a person is dilatory or inclined to dodge payment, he gets his bill each month with the accumulations, and after a fixed time "please remit" appears on the bill. Then, if not paid, it goes to a collector. Of the callous few who fall into the collector's hands, the majority succumb to his importunities.

All this is done as a matter of routine, being entirely in the hands of the bookkeeper, and if a personal friend, having fallen into careless ways, falls into the hands of the collector, his wounded feelings are soothed by being told he is a victim of the "System." He generally pays the bill, however.

To save clerical work, the account of the preceding month is rendered in a lump sum, but itemized accounts with full details are given to those who for any reason question the statement.

The third secretary is general utility. She answers the telephone, marks all the diagram cards, which, by the system of colored pencils and hieroglyphics, show at a glance complete details of all work done; orders all supplies and approves bills for same for the treasurer to pay; conducts all correspondence not specially related to appointments.

In the last half hour of the day she comes to a desk back of my chair, and, calling off the names of the day's patients, writes from my dictation a complete record of the work done and the fee. Patients taking the last hour expect this kind of interruption. As the fees are all given in a code,listeners are none the wiser.

From the differentjournals on which such records are are none the wiser.

From the different journals on which such records are written she marks the diagram cards.

CHAIR ASSISTANTS.

There is one assistant for every two operators, with the exception of one, who, by special arrangement, has one assistant to himself. As each assistant works only on one floor, it is quite easy to take care of two operators.

The assistant sterilizes the instruments and puts them away; gives help in operating when needed, although this is not encouraged as a rule. The patients in our office do not care to have a young woman of the laity looking into their mouths. There is a feeling of privacy about their affairs which makes them want only one person, and that a professional one, studying their infirmities. The assistant keeps notes of supplies and memoranda of such as need replenishing. She makes out the order slips (sample shown) for the laboratories, which are used by the laboratory assistants as a guide to their work and to send to the bookkeeper later on for her to make the charges. The laboratory assistant meantime has marked on the slip the cost of material used and the time spent in the work.

LABORATORY ASSISTANTS.

One is a man formerly a manufacturing jeweler. He does the plate work, bridge work, etc., but does not, of course, see patients.

The other assistant is a woman who does the porcelain work—making inlays, porcelain crowns, continuous gum, etc. The generally accepted belief that a woman can excel a man in matching ribbons, applies to the matching of porcelains, and makes a woman particularly successful in this department.

It is the intention to keep the equipment of the laboratories thoroughly up-to-date.

The housekeeper has charge of the janitor and laundress, looks to the upkeep of the house appurtenances, and dusts the house twice every twenty-four hours.

The janitor sweeps the house from top to bottom every day, and does the other work pertaining to his position. In addition, he goes down town three times a week to do the banking and general errands.

The laundress, with an average of——pieces of linen a day, is kept pretty busy, although her burden is much lightened by the use of an electric iron.

The door-boy does the obvious and also runs errands between the offices.

The secretaries and laboratories are all connected by private telephone, saving many steps and much confusion. In my own operating room is a dial, operated from the desk of the secretary on my floor, where is shown by her, pressing the right button or buttons, the fact that a patient has arrived, length of appointment and work to be done. This will soon be extended to the other offices.

There is no time lost in changing patients. As the operator gets toward the end of his work, he presses a button, which calls his assistant. She collects all the instruments which have been used, and as the patient leaves the chair, proceeds to change the head-rest cover and wipe around the cuspidor. In the meantime another electric signal has been sent to the secretary in the reception room, and the outgoing patient meets his successor coming in.

All new patients when asking for appointments are requested to give the name of the one who sent them, and this is recorded on the ledger card. This serves as a sort of voucher and also as a family tree—one can trace back, card to card, to the original patient.

The question of fees is always an interesting one to younger practitioners and also to many of the older ones. It seems to the writer best to fix in one's mind a minimum fee for an hour's work, based on one's experience in practice, and the environment in which he is placed. This stated fee is capable of increase as time and circumstances permit. Having this fixed sum in mind, make a definite charge for each operation. If the total much exceeds your average per hour, then trim it a bit. If it falls far short, raise it, but not necessarily to the full hourly rate. For example, I have by a combination of fortuitous circumstances done in one hour such fillings as a fair charge apiece would make an amount equivalent to two hour's work. I give the patient some credit for the smoothness and rapidity of the work, and split the difference with him, making a charge equivalent to one and one-half hours' work. Perhaps in the very next hour I do an operation for which ordinarily the fee would be equivalent to one-half hour's work. To be sure, the patient has been restless and difficult to work for, but I do not blind myself to the fact that the previous hour's hard sustained effort has not left me in the best condition to control patient number two, and so here again I split the difference with this patient. This strikes a general average, and one feels that he has dealt with justice and equity to both himself and his patient.

While it is undoubtedly true that no good dentist is ever paid quite enogh for his services, it is also true that he must in order to be successful and to hold his practice, conform to the financial standards of the locality in which he practices. In all other specialties a fixed fee is charged to every patient entering the office, however trivial the attention he receives—and the fee generally is not trivial. It seems to the writer that a dentist should take a different position because his practice is different. He does not as a rule deal with sporadic cases, but rather with regular patients, and whole families of them. He should therefore aim to be the family protector and give much time and advice for which there can be no fee charged. For instance, if one of the children in my practice receives a blow while playing and thinks he has loosened or broken a tooth, I want him to feel that I am the first one to run to, and not that he must first go home and see if his parents think it of enough consequence to send him to incur a fee. All such observations, brief attentions, time suggestions, etc., have no fee, ogre-like, hovering above them. For the same reason I make as a rule no charge for putting in wedges.

This general care and fathering of one's big family does not prevent coming down full and hard with a fee when actual operations have been performed. Patients will object to paying a dollar in one situation while cheerfully paying a hundred in the next.

Patients breaking appointments without due notice are charged for the time.

"Time is money" applies strictly to dentists, for they alone never can make up time lost, and it is fair to assume that he who wantonly or carelessly breaks an appointment is spending that time more profitably to him either in pleasure or in making money.

While every associate does at will each and every kind of work that dentists may do, there is a tendency to specialize in orthodontic and prosthetic dentistry, and by general consent one associate does most of the one and another most of the other.

In closing let the writer express the hope that while his paper may not be of value in itself, it will at least be fruitful of a discussion that will shed much light and be of help to those seeking for the best and most practical ways of conducting a practice.—*The Journal.*

